# Deep Genomic Characterization of *Bacillus subtilis* SNUY1 & 168 Using MAYA (Multistage Analysis of Prokaryotic Annotations): An Integrated Snakemake-Based Bioinformatics Pipeline

**DOI:** 10.3390/ijms27209188

**Published:** 2026-10-16

**Authors:** Sastha Kumar Narendran, Anandhu Presannan, Lekshmi Indulekha, Abhijith Shibu, Adithiyakrishnan Rajeev, Satheesh Krishna Chakkadath, Vidhu S. Vijay, Pradeesh Babu, Geetha Kumar, Bipin G. Nair, Sabarinath Subramaniam, Nidheesh Melethadathil

**Affiliations:** 1 Amrita School of Biotechnology, Amrita Vishwa Vidyapeetham, Amritapuri, Kollam 690525, Kerala, India; am.ls.r4bio24426@am.students.amrita.edu (S.K.N.); am.ls.p2bif25001@am.students.amrita.edu (A.S.);; 2 Sivasakthi Science Foundation, Trivandrum 695003, Kerala, India

**Keywords:** snakemake, *Bacillus subtilis*, genome annotation, antimicrobial resistance, biosynthetic gene clusters

## Abstract

*Bacillus subtilis* is an extensively characterized Gram-positive bacterium with broad applications in agriculture, biotechnology, and biocontrol. Strain SNUY1, known for its antifungal properties and use as an edible coating, has been completely sequenced and deposited in NCBI (accession: CP199918), making it an important model for genomic and functional characterization. Here, we present the MAYA pipeline, a fully automated, rule-based Snakemake workflow that integrates eighteen bioinformatic tools into a single end-to-end framework. MAYA accepts either raw Illumina paired-end FASTQ reads or pre-assembled FASTA sequences, utilizing per-rule Conda environment isolation to ensure strict version control and reproducibility across all constituent tools from quality control and assembly through functional annotation, plasmid typing, and detection of biosynthetic and antimicrobial resistance gene clusters. To benchmark pipeline accuracy across both input modes, MAYA was evaluated using *Bacillus subtilis* subsp. *subtilis* str. 168(AL009126 OZ025638). The FASTA assembly yielded a circularized 4,215,606 bp chromosome (N50: 4.21 Mb, 43.51% GC) with 98.28% CheckM completeness and 100.0% BUSCO ortholog recovery. Prokka annotation predicted 4214 CDSs (86 tRNAs, 30 rRNAs) in FASTA mode compared to 4313 CDSs (106 tRNAs, 25 rRNAs) in FASTQ mode, reflecting expected read fragmentation in unassembled data. DIAMOND mapping against SwissProt yielded 4135 hits (99.90% mean identity) in FASTA mode and 4170 hits (99.75% mean identity) in FASTQ mode. Both modes identified identical ST1 Multilocus Sequence Typing (MLST) profiles and six core AMR determinants (*mphK*, *vmlR*, *rphC*, *aadK*, *satA_Bsub*, and *tet(L)*), while antiSMASH detected 15 biosynthetic gene cluster (BGC) regions (22 protoclusters) in FASTA mode versus 16 regions (23 protoclusters) in FASTQ mode. MOB-suite resolved two non-mobilizable plasmids in the FASTQ assembly (bioproject: PRJNA76): pAC670 and a novel 1666 bp plasmid. Furthermore, nucleotide BLASTN analysis resolved a 76-CDS discrepancy between published NCBI annotations and MAYA/Prokka, demonstrating 95.5% (4081/4272) stringent sequence match and confirming that differences stem from gene-model predictions rather than omitted loci. To demonstrate the FASTA-mode application workflow, the complete SNUY1 (NZ_CP199918) genome assembly was processed through MAYA, yielding a single, contiguous chromosome of 4,196,931 base pairs with a GC content of 43.69% (98.28% CheckM, 100% BUSCO completeness). Annotation identified 4196 coding sequences, while antiSMASH profiling revealed 13 candidate biosynthetic gene cluster (BGC) regions comprising 20 protoclusters (including NRPS, lanthipeptides, terpenes, and sactipeptides), aligning with the strain’s biocontrol capabilities. AMR profiling identified six chromosomally encoded resistance genes (*mphK*, *vmlR*, *rphC*, *aadK*, *satA_Bsub*, and *tet(L)*). In silico mobility analysis via MOB-suite demonstrated that these sequences lack functional conjugation machinery and are classified as non-mobilizable. Executing in 3–4 h on an Intel Xeon server (128 GB RAM, 29 CPU cores), MAYA delivers an efficient, audit-ready framework for end-to-end bacterial genomic profiling.

## 1. Introduction

*Bacillus subtilis* is a Gram-positive, rod-shaped, spore-forming organism that is widely found in soils, waters, root nodules, and the digestive systems of humans and other animals [1]. It is widely distributed across soil, water, food matrices, and animal gastrointestinal tracts, and remains one of the most extensively studied Gram-positive bacterial model organisms [2,3]. Beyond its scientific prominence, *Bacillus subtilis* holds notable regional significance. It was designated as the state microbe of Kerala, India, in recognition of its remarkable ecological prevalence across the state’s diverse environmental niches and its established role in traditional fermented food systems. In addition, *Bacillus subtilis* has extensive applications in agriculture as a biocontrol and plant growth-promoting agent, as well as broader contributions to biotechnology and public health [4]. This designation reflects not only the organism’s significance in Kerala’s soil and water ecosystems but also its cultural, agricultural, and economic importance to the state.

*Bacillus subtilis* has become the model system for studies of Gram-positive bacteria [5]. It produces secondary metabolites such as lipopeptides, polyketides, siderophores, bacteriocins, and volatiles that contribute to its antimicrobial properties, adaptability to the environment, and biocontrol potential [6,7]. The genomic characterization of novel *Bacillus subtilis* strains therefore represents both a scientific and applied challenge, requiring integration of assembly, annotation, resistance profiling, and secondary metabolism prediction tools into a single coherent analytical framework.

Tools such as Snakemake have become a necessity to execute advanced bioinformatics analysis, which consists of several processing steps [8]. These bioinformatics analyses usually entail the use of many software tools whose output files act as the input files for the succeeding step in the process. By handling the dependencies among different rules in a workflow, Snakemake offers a reliable framework for bioinformatics analyses, ensuring portability, reproducibility, and scalability [9]. Snakemake automates dependency resolution between rules and scheduling of jobs accordingly [10].

While several automated bacterial genome analysis pipelines have been reported, including Bactopia [11], BAGEP [12], nf-core/bacass [13], and the recently published BacExplorer [14], none comprehensively integrates secondary metabolite biosynthetic gene cluster prediction, protein domain annotation, KEGG orthology assignment, k-mer frequency analysis, and tandem repeat identification alongside quality control, assembly, and resistance profiling within a single Snakemake-based reproducible framework. While BacExplorer supports dual FASTQ/FASTA inputs, it lacks key functional and structural modules such as antiSMASH, HMMER, KofamScan, BUSCO, TRF, and Jellyfish. Similarly, Bactopia provides extensive modularity; it relies on Nextflow and does not incorporate secondary metabolite prediction. MAYA bridges these gaps by unifying eighteen analytical tools spanning functional genomics, secondary metabolism, and genome quality assessment into a single, reproducible, Conda-managed Snakemake workflow. Capable of processing both raw Illumina reads (FASTQ) and pre-assembled sequences (FASTA), MAYA delivers an end-to-end analysis that encompasses genome assembly, functional annotation, AMR profiling, and secondary metabolite prediction. To demonstrate its utility, *Bacillus subtilis* SNUY1 (NZ_CP199918) was selected as a case-study organism, motivated by its demonstrated antifungal properties, its application as an edible coating [15], and its designation as the state microbe of Kerala, India, reflecting its key ecological, agricultural, and economic significance.

## 2. Results

### 2.1. Genome Assembly and Quality Assessment

Analysis of the assembled genome sequence of *Bacillus subtilis* SNUY1 (NZ_CP199918) using MAYA yielded a single-contig genome assembly of 4,196,931 bp with a GC content of 43.69%, consistent with the known genome characteristics of the *Bacillus subtilis* reference strain [15]. Table 1 summarizes the QUAST assembly statistics of *Bacillus subtilis* strain SNUY1 (NZ_CP199918). The assembly consisted of a single contig with a total genome length of 4,196,931 bp, an N50 value of 4,196,931 bp, and a GC content of 43.69%, indicating a high-quality and complete genome assembly Figure 1.

CheckM-based assessment using the Bacteria taxonomy workflow confirmed 98.8% genome completeness, 0.00% contamination, and zero strain heterogeneity Figure 1. BUSCO analysis against the bacteria_odb10 lineage dataset demonstrated 100% genome completeness for *Bacillus subtilis* SNUY1 (NZ_CP199918), with all 124 BUSCO groups identified as complete and single copy. No fragmented or missing BUSCOs were detected Figure 2.

### 2.2. Prokka Structural Annotation

Structural annotation using Prokka identified 4196 coding sequences (CDS) in the *Bacillus subtilis* SNUY1 (NZ_CP199918) assembly. In addition to protein-coding genes, Prokka predicted 30 rRNA, 86 tRNA, and 1 tmRNA. This annotation served as the primary input for downstream protein-level functional analyses, including DIAMOND, HMMER, and KofamScan. There are major discrepancies in the number of CDS in the genome compared to the number reported by NCBI PGAP, with a difference of 76 genes (1.8%) Figure 3.

### 2.3. Functional Annotation

Protein-level functional annotation was carried out through three complementary approaches. DIAMOND BLASTP searches against the SwissProt database revealed high-confidence annotation of predicted proteins in *Bacillus subtilis* SNUY1 (NZ_CP199918), with strong homology to proteins involved in replication, transcription, translation, and metabolism [16]. HMMER-based hmmscan against Pfam-A identified several conserved domains associated with ATPase activity, DNA replication, helicase function, and transcriptional regulation. Multiple AAA-family ATPase domains and DNA polymerase III-associated domains were detected, indicating active genome maintenance, energy metabolism, and cellular regulatory mechanisms. KofamScan-based KEGG Orthology (KO) assignment revealed genes involved in replication, transport, metabolism, transcriptional regulation, and cellular maintenance [17] Figure 4.

### 2.4. Resistance Gene Detection and Multilocus Sequence Typing

ABRicate search using curated antimicrobial resistance gene databases found several antimicrobial resistance-related genes within the genome of the isolated *Bacillus subtilis* SNUY1 (NZ_CP199918), such as those encoding macrolide resistance, rifamycin resistance, aminoglycoside resistance, streptothricin resistance, and tetracycline resistance. Genes encoding resistance determinants, including *mphK*, *vmlR*, *rphC*, *aadK*, *satA_Bsub*, and *tet(L)*, were identified using the NCBI AMRFinderPlus database with a minimum identity threshold of 80% and a minimum coverage threshold of 80%. All six genes were located on the single assembled chromosomal contig and belong to gene families broadly reported as chromosomally encoded, intrinsic resistance elements within the Bacillus genus, generally lacking association with mobile genetic elements [18]. Consistent with the broader literature on intrinsic resistance in non-pathogenic environmental bacteria such as *Bacillus subtilis*, these determinants are generally considered non-transmissible and unlikely to contribute meaningfully to clinical AMR burden [19]. However, no plasmid replicon screening or mobile-element flanking-region analysis was performed in this study to confirm this directly for SNUY1 (NZ_CP199918); this assessment should therefore be regarded as consistent with genus-level literature rather than a strain-specific finding. Multilocus Sequence Typing (MLST) based on the allelic profiles of the seven housekeeping genes glpF(1), ilvD(1), pta(36), purH(66), pycA(26), rpoD(1), and tpiA(1) revealed that *Bacillus subtilis* SNUY1 (NZ_CP199918) did not match any existing sequence type in the PubMLST database, yielding a novel allelic profile consistent with its species classification. This Multilocus Sequence Typing (MLST) result provides strain-level sequence typing information only; genome assembly completeness and integrity are supported independently by the BUSCO and CheckM results reported above Figure 5.

### 2.5. Secondary Metabolite Cluster Prediction

Secondary metabolite biosynthetic gene clusters (BGCs) were predicted in the *Bacillus subtilis* SNUY1 (NZ_CP199918) genome using antiSMASH with Prodigal-based gene-finding. antiSMASH analysis identified 13 candidate biosynthetic gene cluster regions, comprising 20 individual protoclusters, in the *Bacillus subtilis* SNUY1 (NZ_CP199918) genome. The predicted clusters included lanthipeptides, non-ribosomal peptide synthetases (NRPS), terpenes, trans-AT polyketide synthases (transAT-PKS), type III polyketide synthases (T3PKS), beta-lactone (beta-lactone-containing clusters), CDPS (cyclodipeptide synthase clusters), sactipeptide, and metallophore-associated clusters [6]. These biosynthetic regions suggest biosynthetic potential for antimicrobial and antifungal metabolite production consistent with the well-documented biocontrol properties of *Bacillus subtilis* [7]; however, experimental validation is required to confirm the activity of specific metabolites in this strain.

### 2.6. tRNA, rRNA, and Repeat Elements

tRNA genes were cataloged using tRNAscan-SE. Analysis identified multiple tRNA genes distributed throughout the *Bacillus subtilis* SNUY1 (NZ_CP199918) genome. Various amino acid-specific tRNAs, including tRNAs for alanine, leucine, lysine, arginine, glycine, and serine, were detected with high prediction scores. A summary of tRNA gene predictions is provided in Table 2.

rRNA genes were identified using Barrnap, contributing to a total of 30 ribosomal RNA genes in the *Bacillus subtilis* SNUY1 (NZ_CP199918) genome, including multiple copies of 5S, 16S, and 23S rRNA genes. The Tandem Repeat Finder (TRF) analysis identified multiple tandem repeat regions distributed across the *Bacillus subtilis* SNUY1 (NZ_CP199918) genome. Several repetitive DNA motifs with varying copy numbers and repeat lengths were detected, indicating the presence of conserved repetitive elements that may affect genome organization and stability.

### 2.7. Pipeline Benchmarking and Validation Using Model Strain 168

The assembly pipeline yielded a complete chromosome for *Bacillus subtilis* subsp. *subtilis* str. 168 (AL009126 OZ025638). QUAST metrics confirmed that the genome assembled into a single contiguous sequence of 4,215,606 bp with an N50 of 4,215,606 bp (L50 = 1), a 43.51% GC content, and zero gap regions. CheckM analysis verified strong lineage-specific accuracy, scoring 98.28% completeness with 0.00% contamination and no strain heterogeneity. Annotation predicted 4226 genes with an 87.89% coding density using NCBI Translation Table 3. Finally, BUSCO benchmarking against the bacterial dataset (bacteria_odb10) achieved a perfect 100.0% score for complete, single-copy orthologs with no missing or fragmented genes (C: 100.0% [S: 100.0%, D: 0.0%], F: 0.0%, M: 0.0%). Taken together, these results confirm a high-fidelity, circularized bacterial genome free of sequence gaps or sample contamination.

### 2.8. Prokka Structural Annotation for Strain 168

FASTA:

Structural annotation using Prokka identified 4214 coding sequences (CDS) in the *B. subtilis* subsp. *subtilis* str. 168 (AL009126 OZ025638) assembly. In addition to protein-coding genes, Prokka predicted 30 rRNA, 87 tRNA, and 1 tmRNA. This annotation served as the primary input for downstream protein-level functional analyses including DIAMOND, HMMER, and KofamScan Table 4.

FASTQ:

Structural annotation using Prokka identified 4313 coding sequences (CDS) in *Bacillus* *subtilis* subsp. *subtilis* str. 168 (bioproject: PRJNA740103) assembly generated from the raw FASTQ sequencing dataset. In addition to protein-coding genes, Prokka predicted 25 rRNA, 106 tRNA, and 1 tmRNA Table 4.

### 2.9. Functional Annotation for Strain 168

FASTA:

DIAMOND BLASTP analysis of the assembled genome against the SwissProt database mapped 4135 unique protein-coding sequences. The assembly exhibited near-perfect sequence identity (mean: 99.90%) with an average alignment length of 295.9 amino acids. Statistical confidence was exceptionally high across predicted proteins, yielding a median E-value of 2.16 × 10^−180^ and bit scores ranging from 36.2 to 10,851 Table 5.

FASTQ:

Direct functional profiling of the read dataset (bioproject: PRJNA740103) yielded 4170 unique query hits against SwissProt. Sequence conservation remained consistently strong, showing a mean identity of 99.75% and an average alignment length of 293.6 amino acids. High-confidence alignments were maintained (median E-value: 2.48 × 10^−179^; bit scores: 36.2–10,015). The slightly higher hit count and marginal reduction in alignment length reflect expected read fragmentation within unassembled sequencing data Table 5.

### 2.10. Resistance Gene Detection and Multilocus Sequence Typing for Strain 168

ABRicate analysis within the MAYA pipeline using the NCBI AMRFinderPlus database identified six AMR-related genes (mphK, vmlR, rphC, aadK, satA_Bsub, and tet(L)) in the assembly FASTA dataset (GCF_000009045.1_ASM904v1_genomic). The FASTQ dataset (fastq_SRR15006059, bioproject: PRJNA740103) recovered the same core profile, with an additional cat-TC hit, while tet(L) was split across two contigs. All identified determinants were detected using ≥80% identity and coverage thresholds Table 6.

Multilocus Sequence Typing (MLST) analysis through MAYA identified an identical ST1 profile in both FASTA and FASTQ datasets. All seven housekeeping loci (glpF, ilvD, pta, purH, pycA, rpoD, and tpiA) showed 100% identity, supporting the assignment of *Bacillus subtilis* 168 (AL009126 OZ025638) to ST1 Table 7.

### 2.11. Secondary Metabolite Cluster Prediction for Strain 168

FASTA: antiSMASH identified **15 candidate Biosynthetic Gene Cluster (BGC) regions comprising 22 protoclusters** across the single assembled chromosome (NC_000964.3). The predicted clusters included sactipeptides, ranthipeptides, non-ribosomal peptide synthetases (NRPS), trans-AT polyketide synthases (transAT-PKS), type III polyketide synthases (T3PKS), terpenes and terpene precursors, PKS-like clusters, glycocins, beta-lactones, cyclodipeptide synthases (CDPS), RRE-containing clusters, epipeptides, and NRP-metallophore-associated clusters Table 8.

FASTQ: antiSMASH identified **16 candidate BGC regions comprising 23 protoclusters** distributed across four assembly contigs (NODE_1, NODE_2, NODE_5, and NODE_6). The additional region and protocluster detected in the FASTQ-derived assembly reflect the fragmented nature of the read-based assembly Table 8.

### 2.12. tRNA, rRNA, and Repeat Elements for Strain 168

The tRNA genes were identified using the tRNAscan-SE module integrated within the MAYA pipeline. Analysis of the *Bacillus subtilis* 168 (AL009126 OZ025638) reference genome (FASTA) identified 86 tRNA genes, while the FASTQ-derived assembly (fastq_SRR15006059) contained 114 predicted tRNAs, including 105 standard tRNAs, 4 pseudogenes, and 5 undetermined tRNAs. The major amino acid classes detected in both datasets included leucine, glycine, arginine, alanine, valine, threonine, asparagine, and glutamate, with additional tRNA types also identified (Table 9).

### 2.13. In Silico Mobility and Plasmid Profiling of Reference and Novel Constructs for Strain 168

Mobsuit was run to identify the presence of plasmids and plasmid mobility, novel plasmid(novel_21d5243d86abc7218238d2dc65ced188) belonging to Node14 of length 1666 nucleotides were identified when run against fastq file of *Bacillus subtilis* subsp *subtilis* strain 168 (AL009126 OZ025638), Plasmid (AC670), plasmid sequence belonging to Node 12 was detected by mobsuit which codes for tetracycline resistant gene as detected by abricate. Both plasmids are categorized as non-mobilizable, with the AC670 plasmid matching accession number (U32369), which isfound in multiple phyla, including Bacillota, and Pseudomonadota, and the novel plasmid (novel_21d5243d86abc7218238d2dc65ced188) that is also non-mobilizable, with a broad phyla host range like pAC670. Both plasmids show an absence of relaxase/ MPF/oriT and a GC% of 36.28% and 43.64% for AC670 and the novel plasmid, respectively Table 10.

### 2.14. Investigation of the CDS Discrepancy Between NCBI and MAYA/Prokka Annotations

MAYA/Prokka identified 4196 CDSs, whereas the published NCBI annotation contained 4272 CDSs, representing a difference of 76 CDSs (1.8%). To investigate whether this discrepancy represented genuinely missed coding regions or differences in gene-model prediction, we performed a nucleotide-level comparison of the 4272 NCBI-annotated CDSs against the MAYA/Prokka nucleotide sequences using BLASTN. Using a stringent criterion of ≥95% nucleotide identity, ≥90% query coverage, and an E-value ≤ 1 × 10^−10^, 4081 NCBI CDSs (95.5%) showed strong nucleotide-level correspondence with MAYA/Prokka predictions. A further 73 CDSs had detectable BLASTN matches but did not satisfy all the stringent criteria, whereas 118 CDSs had no detectable BLASTN hit under these search conditions. Thus, the observed difference of 76 CDSs between the two annotations does not correspond directly to 76 BLASTN-negative sequences and should not be interpreted as evidence that 76 genes were necessarily missed by the MAYA/Prokka pipeline. The detailed results of the nucleotide-level comparison between the NCBI and MAYA/Prokka CDS annotations are provided in the separately attached Supplementary Table.

### 2.15. Comparative Feature Analysis of MAYA Against Existing Bacterial Annotation Pipelines

While standard workflows such as nf-core/bacass and BacExplorer primarily focus on initial assembly and baseline annotation (e.g., via Prokka or Bakta), MAYA incorporates a multi-layered proteome annotation architecture combining Prokka, DIAMOND BLASTP (SwissProt), HMMER (Pfam-A), and KofamScan (KEGG Orthology). Furthermore, MAYA natively integrates specialized downstream analyses—including antiSMASH (v7.0.0) for biosynthetic gene cluster (BGC) discovery and MOB-suite (v3.1.0) for plasmid reconstruction and mobility profiling—without requiring external plugins or post hoc command-line execution. From a software engineering and reproducibility standpoint, MAYA leverages the Snakemake workflow manager with rule-specific Conda environment isolation, ensuring software version locking across all constituent modules. Additionally, MAYA natively supports dual-input modes (raw paired-end FASTQ reads and pre-assembled FASTA sequences), offering broader utility than single-input tools like BAGEP (FASTA-only) or nf-core/bacass (FASTQ-primary).

## 3. Discussion

The MAYA pipeline serves as an example showing how a workflow structure based on the Snakemake framework can automate the entire process of genomic analysis of bacteria from raw sequencing data all the way to functional annotation [9]. The incorporation of eighteen modules into a single workflow that tracks dependencies addresses the reproducibility challenge in bacterial genomics, where multi-tool workflows are difficult to manage and standardize [20].

As summarized in Table 11, the MAYA pipeline assembled *Bacillus subtilis* SNUY1 (NZ_CP199918) into a single contig of 4,196,931 bp with a GC content of 43.69%, which is comparable to the published SNUY1 (NZ_CP199918) genome report showing an identical genome size and a GC content of 43.7%. The Prokka-derived annotation yielded 4196 CDS, 86 tRNA genes, 1tmRNA, and 30 rRNA genes. The CDS count differs slightly from the 4272 CDS reported in the published genome (Table 12); this discrepancy most likely reflects differences in annotation tool versions and gene prediction thresholds between Prokka and the original NCBI annotation pipeline. Furthermore, no ncRNAs were detected by Prokka compared with the 5ncRNAs detected in the NCBI annotation; this discrepancy is consistent with known inter-tool variation in prokaryotic gene prediction. The tRNA and rRNA counts matched exactly, supporting the overall completeness and accuracy of the assembly [15].

The dual-mode design of the MAYA pipeline, capable of accepting both FASTQ and FASTA inputs using the spades_fastq and fasta_copy rules, respectively, greatly improves its applicability for microbial genome studies. In this way, the pipeline can process both raw sequencing reads and assembled genome sequences, which caters to various needs and workflows of users. The capability to handle several input formats helps improve workflow flexibility, minimize the need for preprocessing steps, and efficiently integrate datasets from various sequencing platforms and assembly approaches. In the present study, only the FASTA-mode pathway was executed and validated using the complete genome of *B. subtilis* SNUY1 (NZ_CP199918); the FASTQ-mode pathway was additionally validated using publicly available raw sequencing reads of the well-characterized *Bacillus subtilis* strain 168 (SRA accession: SRR15006059, bioproject: PRJNA740103). The FASTQ dataset was processed through the relevant raw-read modules, including FastQC, fastp, SPAdes, and Jellyfish, followed by the downstream genomic analysis modules of MAYA. The corresponding FASTA- and FASTQ-based results are presented in Section 2.7, Section 2.8, Section 2.9, Section 2.10, Section 2.11, Section 2.12, Section 2.13, Section 2.14 and Section 2.15, providing validation of both input modes of the pipeline.

The integration of antiSMASH for secondary metabolite cluster prediction is of relevance for *Bacillus subtilis*, a taxon known for its rich biosynthetic repertoire [6]. The combined use of Prokka, DIAMOND, HMMER, and KofamScan provides multi-layered functional coverage that is more comprehensive than any single annotation approach, enabling both broad functional classification and fine-grained domain-level analysis of the predicted proteome.

The difference in CDS number between the MAYA/Prokka and NCBI annotations does not necessarily indicate systematic loss of coding sequences by the MAYA pipeline. The nucleotide-level comparison showed strong correspondence for the majority of NCBI CDSs, suggesting that part of the observed difference may arise from differences in gene-model boundaries and annotation criteria between the two approaches. Nevertheless, the remaining discrepant CDSs warrant consideration when interpreting differences between automated annotation pipelines.

The biosynthetic gene cluster hotspot identified at 1.78–2.06 Mbp in SNUY1 (NZ_CP199918) is particularly noteworthy. This region harbors overlapping transAT-PKS, PKS-like, T3PKS, NRPS, and betalactone clusters, suggesting a concentrated biosynthetic capacity that likely contributes to the strain’s known antifungal properties [15]. The presence of multiple NRPS and PKS clusters aligns with the well-documented role of *Bacillus subtilis* lipopeptides, such as surfactin, iturin, and fengycin, in plant disease biocontrol [7].

MAYA’s Conda environment manager, which employs environment definition per rule, allows for tool version isolation and enables environment-based reproducibility, addressing version-specific differences that affect downstream interpretations [9,20]. Snakemake’s automatic rule re-running further supports robust data processing.

The Multilocus Sequence Typing (MLST) analysis further provided a molecular characterization of SNUY1 (NZ_CP199918). The novel Multilocus Sequence Typing (MLST) profile, which does not match any existing sequence type in the PubMLST database, provides a unique molecular identifier for this strain and may be valuable for strain tracking in commercial biocontrol formulations.

This study represents a proof-of-concept validation of the MAYA pipeline performed on a single bacterial genome (*Bacillus subtilis* SNUY1 (NZ_CP199918)) and provides biological insight; future work will extend this evaluation to multiple bacterial species and additional sequencing datasets to establish broader generalizability and robustness. The present limitations of the MAYA pipeline are its inability to work with assemblies generated from long-read and hybrid assembly methods [21,22]. Future developments should aim at supporting long-read and hybrid assembly algorithms such as Flye and Unicycler [23], as well as implementing modules for plasmid analysis and comparative genomics. Additionally, the current pipeline does not include comparative genomics modules such as OrthoFinder-based pan-genome analysis [24] or MASH-based species-level phylotyping [25], which would further contextualize isolate characterization within broader genomic diversity frameworks.

Validation of the MAYA pipeline was conducted using the golden reference strain *Bacillus subtilis* subsp. *subtilis* str. 168 (AL009126 OZ025638), evaluating the workflow’s efficiency and benchmarking its automated output against a highly curated genomic baseline. While MAYA provides a robust and audit-ready analytical framework, certain operational boundaries apply to its current release. Notably, input processing is restricted to short-read Illumina paired-end FASTQ or pre-assembled FASTA sequences, requiring manual external assembly for long-read Oxford Nanopore or PacBio datasets prior to analysis. Furthermore, because MAYA concurrently executes memory-intensive modules—including antiSMASH, KofamScan, and HMMER—multi-threaded or batch executions demand substantial computational resources, requiring dedicated CPU allocation and high-memory environments for optimal performance.

## 4. Materials and Methods

### 4.1. Genome Data Retrieval and Input Configuration

The assembled genome sequence of *Bacillus subtilis* SNUY1 (NZ_CP199918) was used as the primary input for the MAYA pipeline, retrieved from the NCBI Genbank database. The assembled genome FASTA file is stored in the data/directory of the MAYA pipeline following the predefined input organization structure for reproducible workflow execution.

### 4.2. Pipeline Architecture and Workflow Management

The MAYA pipeline was developed using the Snakemake workflow management system [9], in which each analytical module is encoded as an independent rule with explicitly defined inputs, outputs, Conda environment specifications, and shell commands. The pipeline accepts two types of inputs: (i) FASTQ input mode, where raw reads are subjected to quality control and assembled de novo; and (ii) FASTA input mode, where the assembled contig sequences are simply copied to the output assembly (results/spades/{sample}/contigs.fasta) using the rule fasta_copy. Sample sets are specified at the beginning of the Snakefile, and FASTQ_SAMPLES and FASTA_SAMPLES are considered separate lists that are combined into one SAMPLES list. The main rule, rule all, specifies all output files expected from the workflow, allowing Snakemake to automatically resolve workflow dependencies and execute the complete workflow.

All bioinformatic steps were orchestrated using MAYA, a Snakemake-based workflow utilizing rule-specific Conda environment isolation to guarantee software version locking and reproducibility. Quality control and trimming were performed using FastQC (v0.12.1) and Trimmomatic (v0.39). Genome assembly assessment was conducted via CheckM (v1.2.2) and BUSCO (v5.5.0). Functional and structural RNA annotations were generated using Prokka (v1.14.6), with dedicated RNA locus calls generated using Barrnap (v0.9) and tRNAscan-SE (v2.0.12). Multilocus Sequence Typing and antimicrobial resistance profiling were executed using Multilocus Sequence Typing (MLST) (v2.23.0) and ABRicate (v1.0.1) against the NCBI AMRFinderPlus database. Secondary metabolite gene clusters and plasmid mobility were characterized using antiSMASH (v7.0) and MOB-suite (v3.1.0), respectively.

The pipeline was validated using the FASTQ and FASTA datasets of *Bacillus subtilis* strain 168 (AL009126 OZ025638). Benchmarking was performed on an Intel Xeon i7 server with 128 GB RAM. The complete workflow was executed in approximately 3–4 h, operating at full capacity across 29 CPU cores out of a total of 40 cores. These results demonstrate the pipeline’s computational efficiency and robust handling of high-throughput genomic data.

### 4.3. Database Preparation and Initialization

To ensure reproducibility and facilitate seamless execution for first-time users, the MAYA pipeline requires a localized setup of several large reference databases. Users can automate this setup using the provided scripts/download_databases.sh script, or execute the following steps manually within the databases/directory:

Pfam-A Database (v35.0): The profile HMM database was downloaded from InterPro FTP and indexed using HMMER (v3.3.2):

wget ftp://ftp.ebi.ac.uk/pub/databases/Pfam/releases/Pfam35.0/Pfam-A.hmm.gz -P databases/ gunzip databases/Pfam-A.hmm.gz hmmpress databases/Pfam-A.hmm.

SwissProt/UniProtKB Database: The SwissProt reference proteome was downloaded in FASTA format and indexed using DIAMOND (v2.1.8):

wget ftp://ftp.uniprot.org/pub/databases/uniprot/current_release/knowledgebase/complete/uniprot_sprot.fasta.gz -P databases/ gunzip databases/uniprot_sprot.fasta.gz diamond makedb --in databases/uniprot_sprot.fasta -d databases/swissprot.

AMR and Plasmid Databases: NCBI AMRFinderPlus databases for ABRicate (v1.0.1) and MOB-suite (v3.1.0) reference databases were initialized using amrfinder -u and mob_init, respectively.

### 4.4. Assembly Quality Assessment

Assembly statistics were calculated using QUAST [26] and included the total number of contigs, total assembly size, N50 and L50, as well as the percentage of the assembly consisting of sequences larger than some minimum size. The results of QUAST were reported in HTML reports at results/quast/{sample}/report.html.

The degree of completeness and contamination of the genome was calculated independently using CheckM [27] with the domain-specific Taxonomy workflow targeting the Bacteria domain. This was accomplished using CheckM in long report mode.

Assembly completeness was independently evaluated using BUSCO [28] against the bacteria_odb10 lineage. BUSCO was executed with four threads per sample, and results were written to results/busco/{sample}, providing the proportions of complete single-copy, duplicated, fragmented, and missing conserved orthologs.

All tools were executed within dedicated Conda environments specified per rule, ensuring software version isolation and reproducibility. Dependency resolution and re-execution of incomplete outputs were managed automatically by the Snakemake engine.

### 4.5. Structural and Functional Genome Annotation

#### 4.5.1. Prokka Annotation

The fast structural and functional annotation of the assembly contigs was carried out using the Prokka tool [29] (v1.14.6). This software predicts coding sequences, ribosomal RNA (rRNA), transfer RNA (tRNA), and transfer-messenger RNA (tmRNA) genes, generating outputs in GFF3 format (results/prokka/{sample}/{sample}.gff).

#### 4.5.2. tRNA Gene Prediction

tRNA genes were detected using tRNAscan-SE (v2.0.12) [30] with the bacterial mode option, and the generated results were stored in results/trnascan/{sample}_trna.tsv.

#### 4.5.3. rRNA Gene Detection

Ribosomal RNA genes were detected using Barrnap [31] (v0.9), and GFF3-format output was written to results/barrnap/{sample}/{sample}.gff.

### 4.6. Functional Characterization

#### 4.6.1. Protein Domain Annotation

Domain search for proteins was done using hmmscan [32] from HMMER (v3.3.2) with the Pfam-A database (databases/Pfam-A.hmm). The output format for domains (--domtblout) was stored in results/hmmer/{sample}_pfam.tsv using four CPU threads.

#### 4.6.2. KEGG Orthology Assignment

Functional annotation for the KEGG pathway was done using KofamScan (v1.3.0) [33]. Protein sequences were matched to KO (Kofam database) using HMM-based KEGG Orthology (kofam_db/profiles) and ko_list. This was accomplished using 4 threads.

#### 4.6.3. Protein Similarity Search

Rapid protein-level homology searches were conducted using DIAMOND (v2.1.8) [34] BLASTP against a pre-built SwissProt database (databases/swissprot.dmnd), retaining only the top hit per query (-k 1) with the results written to results/diamond/{sample}.tsv.

### 4.7. Antimicrobial Resistance and Multilocus Sequence Typing MLST

#### 4.7.1. Resistance Gene Screening

Assembled contigs were screened for antimicrobial resistance determinants using ABRicate [35] (v1.0.1), which queries assembled sequences against curated resistance gene databases (NCBI AMRFinderPlus database, ≥80% identity, ≥80% coverage), and the results were stored in results/abricate/{sample}/{sample}. tab. This screening approach follows established AMR pipeline validation practices [36].

#### 4.7.2. Mobsuite

Mobsuite (v3.1.0) was used to identify, reconstruct, cluster and type plasmid sequences from bacterial whole-genome sequencing (WGS) draft assemblies. To predict replicon types, relaxin types, host range and conjugation potential, and to use Mash distance algorithms to cluster related plasmids. Results were obtained and stored in results/mob_recon.

#### 4.7.3. Multilocus Sequence Typing

Multilocus Sequence Typing (MLST) was performed using MLST [37] (v2.23.0) against default PubMLST scheme databases. Results were obtained as results/mlst/{sample}/{sample}.tsv.

### 4.8. Secondary Metabolite and Repeat Analysis

#### 4.8.1. Secondary Metabolite Cluster Prediction

Biosynthetic Gene Clusters (BGCs) were predicted using antiSMASH (v7.0.0) [38] with Prodigal (v2.6.3) as the gene-finding tool (--genefinding-tool prodigal). Output directories were written to results/antiSMASH/{sample}.

#### 4.8.2. Tandem Repeat Identification

Tandem repeats were identified using Tandem Repeats Finder (TRF) (v4.09.1) [39] with default parameters. The FASTA input was temporarily copied, processed, and the resulting .dat file was written to results/trf/{sample}/{sample}.dat.

All tools were executed within dedicated Conda environments specified per rule, ensuring software version isolation and reproducibility. Dependency resolution and re-execution of incomplete outputs were managed automatically by the Snakemake engine.

### 4.9. Nucleotide-Level Comparison of NCBI and MAYA/Prokka CDS Annotations

To investigate differences between the published NCBI annotation and the MAYA/Prokka annotation, the nucleotide sequences of all NCBI-annotated CDSs were extracted from the GenBank file and compared against the nucleotide sequences generated by the MAYA/Prokka pipeline using BLASTN. The NCBI CDSs were used as queries against the MAYA/Prokka nucleotide sequence database. BLASTN hits were evaluated based on nucleotide sequence identity, query coverage, and E-value. A stringent match was defined as ≥95% nucleotide identity, ≥90% query coverage, and an E-value ≤ 1 × 10^−10^.

## 5. Additional Modules for FASTQ Input

The following modules were executed only when raw paired-end FASTQ files were provided as input to the MAYA pipeline. Since the present study utilized an assembled genome sequence in FASTA format, these modules were not executed during the analysis of *Bacillus subtilis* SNUY1 (NZ_CP199918); however, they remain integral components of the workflow.

### 5.1. Read Quality Control

#### 5.1.1. Adapter Trimming and Quality Filtering

The paired-end sequences were analyzed with FastP [40] for adapter removal, quality control, and the elimination of low-complexity sequences. The command-line FastP was run with two threads, and the filtered sequences were saved in compressed FASTQ format (results/fastp/{sample}_1.trim.fastq.gz, results/fastp/{sample}_2.trim.fastq.gz).

#### 5.1.2. Quality Visualization

The trimmed reads were then analyzed via FastQC [41] in order to generate an HTML report for each sample under results/fastqc_trimmed/. The fastqc_trimmed rule had a wildcard condition attached to it so that it only ran on FASTQ-mode samples.

#### 5.1.3. Aggregated Quality Reporting

MultiQC [42] was used to combine all FastQC results into a single HTML report (results/multiqc/multiqc_report.html), making it easier to view and compare sequencing quality metrics across all samples.

### 5.2. De Novo Genome Assembly

SPAdes [43], run in careful mode (--careful flag), which uses the MismatchCorrector tool for post-processing to lower sequencing error rates in the generated contigs, was employed for assembly of trimmed paired-end reads in FASTQ-mode samples. Four processes per sample were used to execute SPAdes, and the output contigs were saved as {sample}/contigs.fasta in results/spades/{sample}. For the FASTA-mode samples, a fasta_copy target was created to generate contigs in the same manner as for the FASTQ-mode.

### 5.3. K-mer Frequency Analysis

K-mer frequency distributions were generated using Jellyfish [44]. Trimmed paired-end reads were decompressed and counted using a k-mer with two threads. The resulting k-mer histogram was written to results/jellyfish/{sample}_histo.txt and subsequently used for genome size estimation and heterozygosity assessment.

## 6. Conclusions

This study developed MAYA, an integrated Snakemake-based pipeline for comprehensive bacterial genome analysis and validated it as a proof-of-concept using the complete genome of *Bacillus subtilis* SNUY1 (NZ_CP199918) retrieved from the NCBI GenBank database. The pipeline successfully automated all stages of genomic characterization and genome completeness assessment, accurately and reproducibly recovering known genomic features from the reference genome. Its modular architecture, Conda environment integration, and dual input-mode support enhance reproducibility, portability, and ease of use across different computational environments. Overall, MAYA provides a practical framework for integrating diverse bioinformatics tools into a unified workflow for bacterial genome characterization.

## Figures and Tables

**Figure 1:**
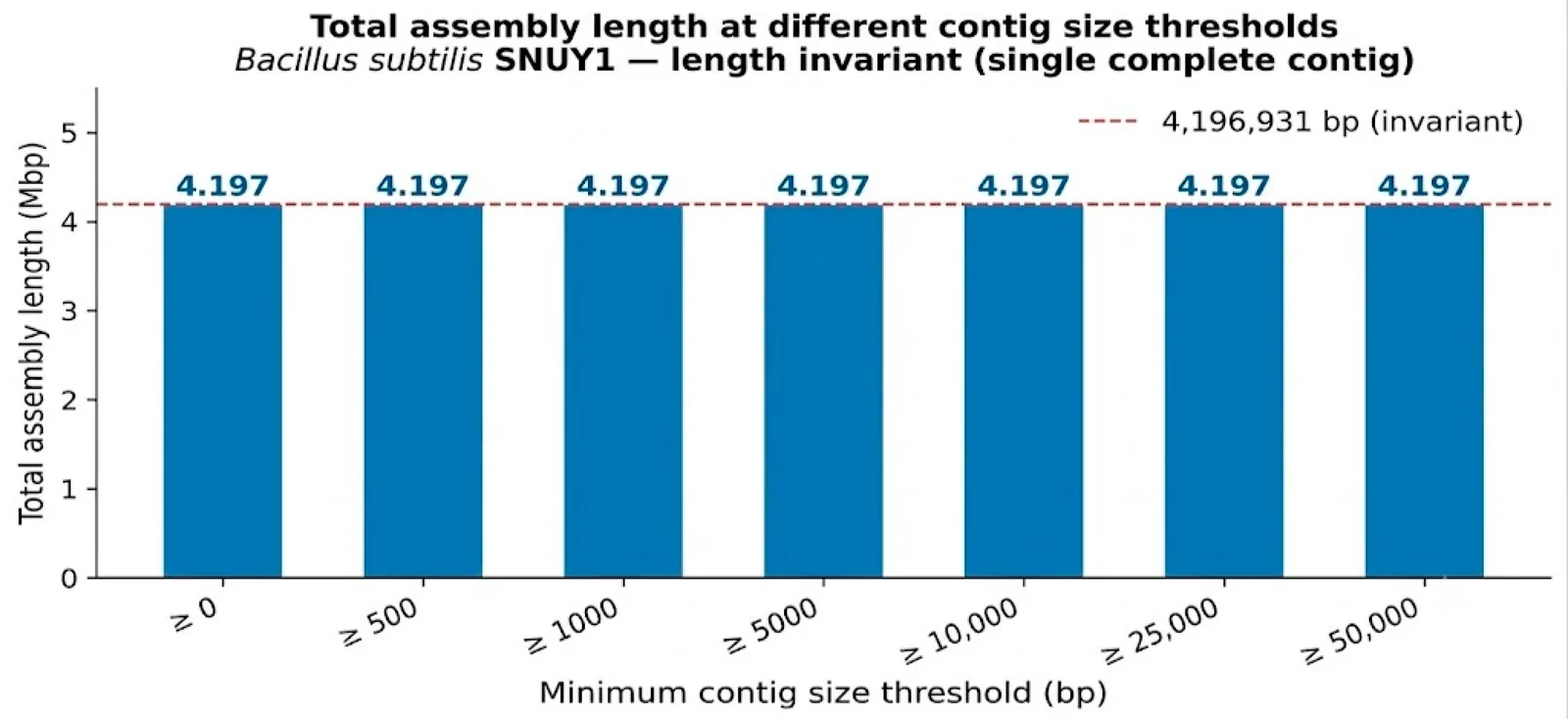
Total assembly length of *Bacillus subtilis* SNUY1 remained constant (4.197 Mbp) across all contig size thresholds, indicating a complete single-contig assembly.

**Figure 2:**
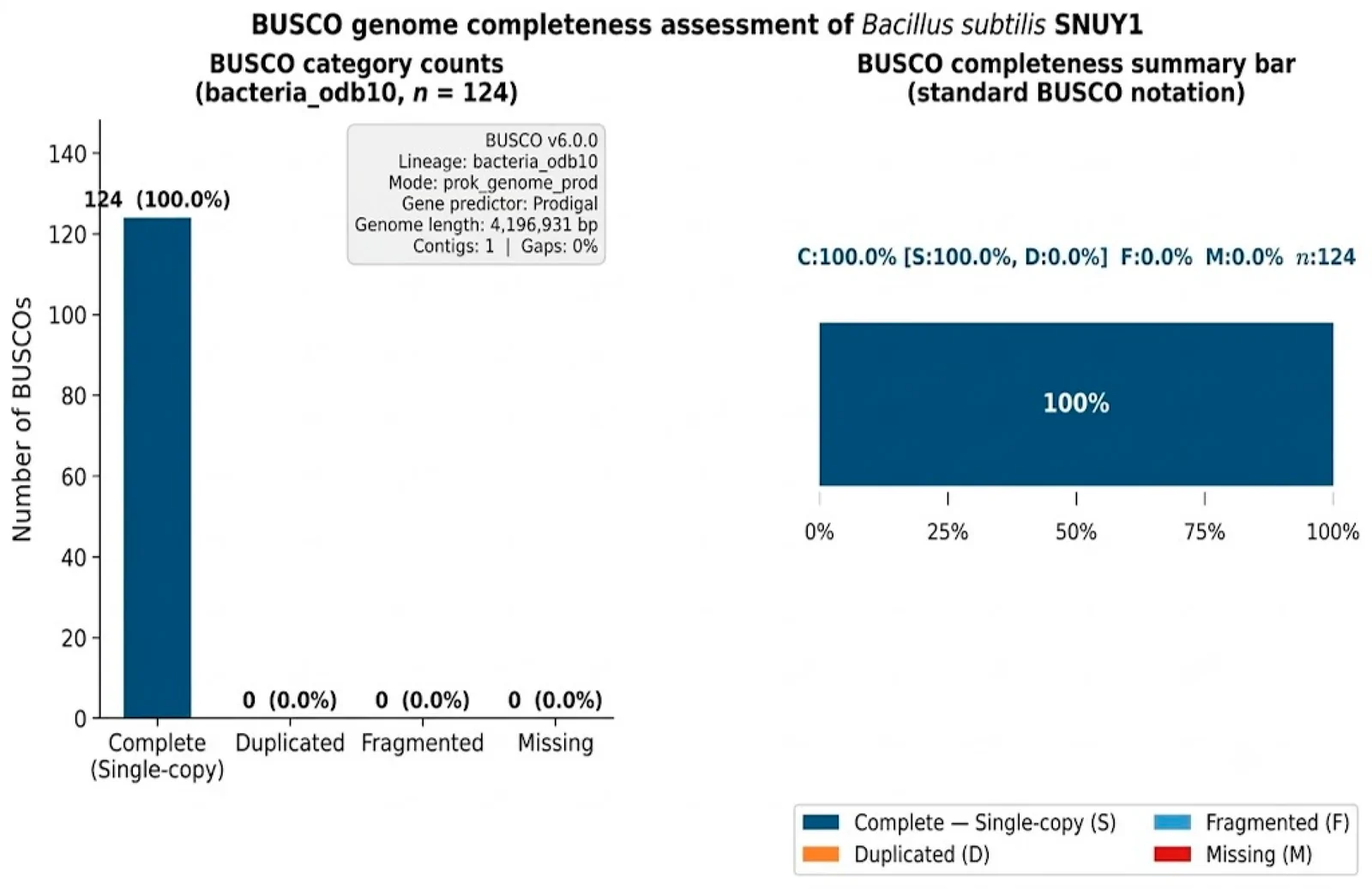
BUSCO genome completeness assessment of *Bacillus subtilis* SNUY1 using the bacteria_odb10 lineage dataset (*n* = 124 BUSCO groups; BUSCO v6.0.0). Bar chart showing counts across the four BUSCO categories. All 124 BUSCO groups were recovered as complete and single copy (C: 100.0%; S: 100.0%), with zero duplicated, fragmented, or missing BUSCOs. Standard BUSCO completeness Summary Bar. The perfect completeness score confirms the high quality and integrity of the assembled genome (4,196,931 bp; 1 contig; 0% gaps).

**Figure 3:**
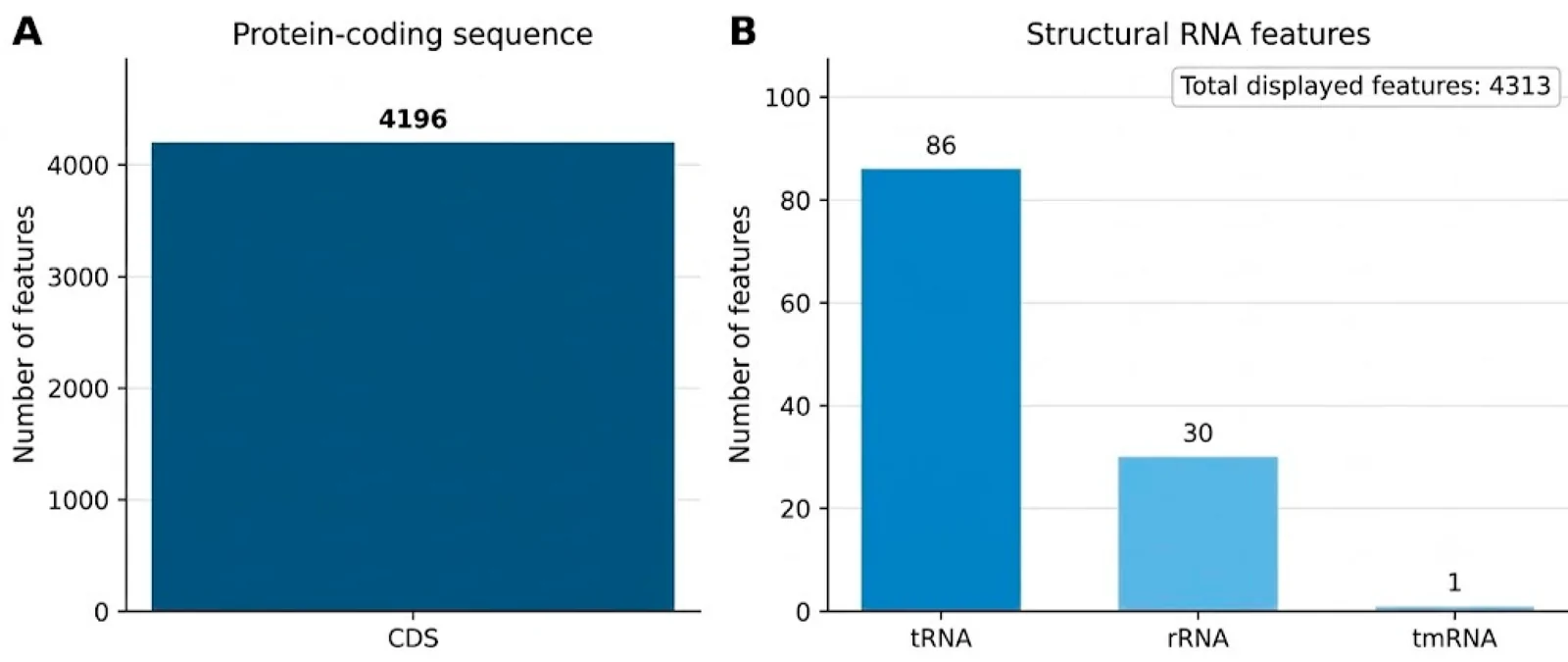
Prokka structural annotation of *Bacillus subtilis* SNUY1. (**A**) Feature counts: CDS (*n* = 4196), tRNA (*n* = 86), rRNA (*n* = 30), and tmRNA (*n* = 1). (**B**) Proportional distribution of RNA gene types (tRNA 73.5%, rRNA 25.6%, tmRNA 0.9%).

**Figure 4:**
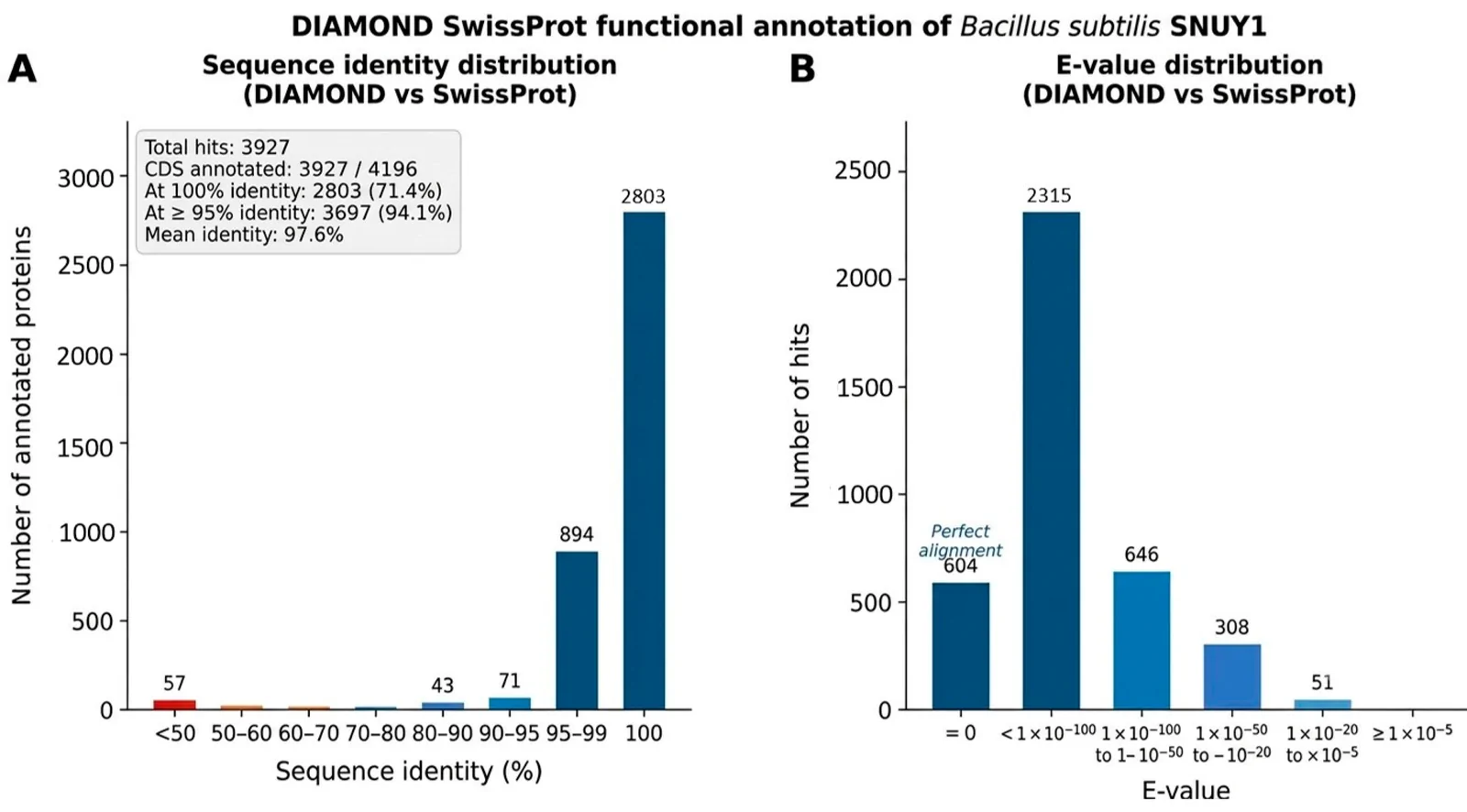
DIAMOND BLASTP functional annotation of *Bacillus subtilis* SNUY1 against the SwissProt database. (**A**) Sequence identity distribution of 3927 annotated proteins. The majority of hits (2803; 71.4%) showed 100% identity to SwissProt entries, and 94.1% of hits had ≥95% identity, reflecting the high sequence conservation of SNUY1 with the *Bacillus subtilis* reference proteome. Mean sequence identity was 97.6%. (**B**) E-value distribution confirming annotation reliability: 604 hits yielded an e-value of zero and 2315 had e-value < 1 × 10^−100^, with only 3 hits exceeding 1 × 10^−5^.

**Figure 5:**
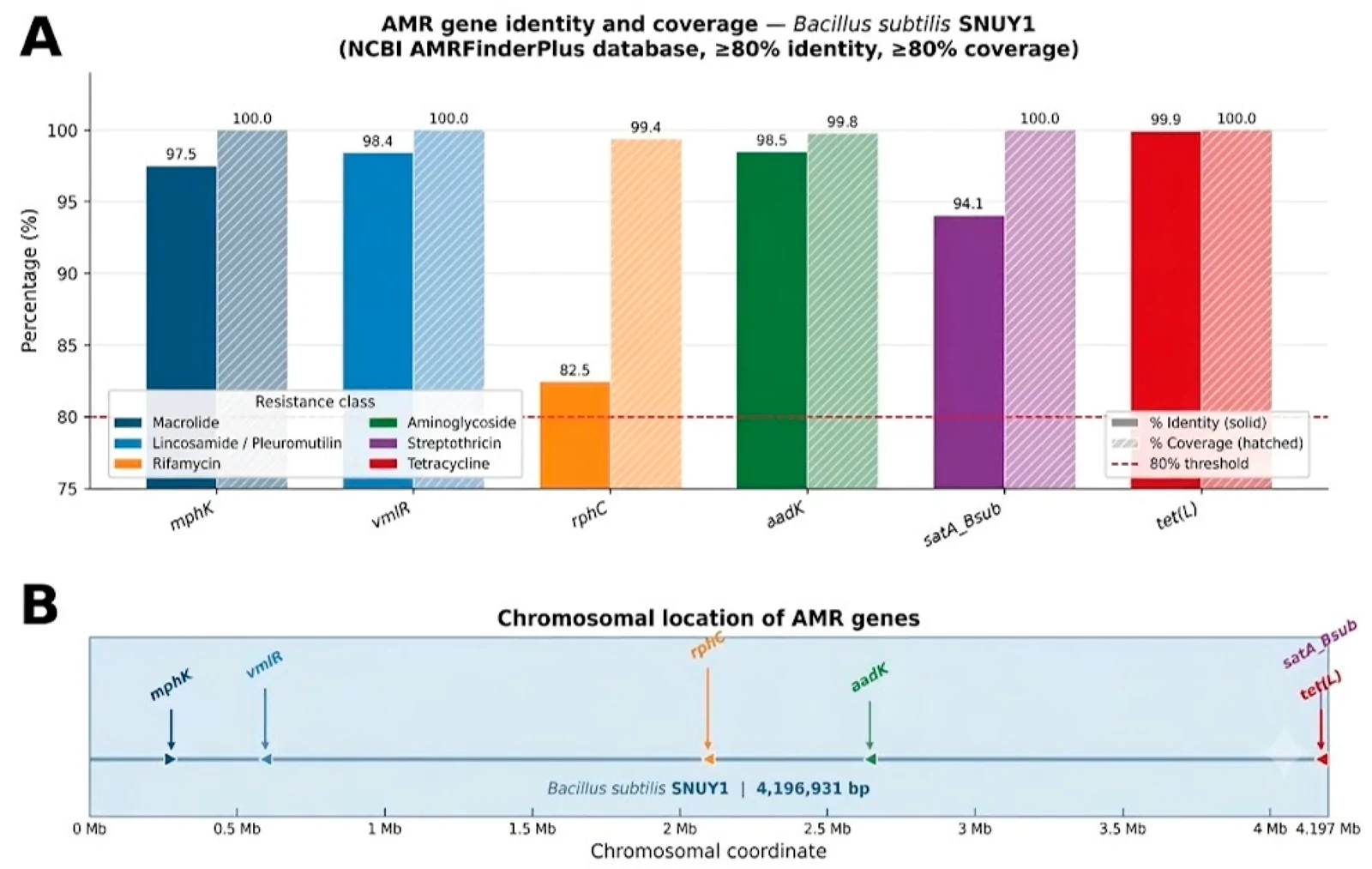
Antimicrobial resistance (AMR) gene profile and genomic localization in *Bacillus subtilis* SNUY1 identified by ABRicate (NCBI AMRFinderPlus database, 80% identity, 80% coverage). (**A**) Grouped bar chart depicting percentage identity (solid bars) and percentage alignment coverage (hatched/slanted line pattern) for each of the six identified AMR genes (*mphK*, *vmlR*, *rphC*, *aadK*, *satA_Bsub*, and *tet(L)*). Bars are color-coded by antibiotic resistance class: dark blue (macrolide), light blue (lincosamide/pleuromutilin), orange (rifamycin), dark green (aminoglycoside), purple (streptothricin), and red (tetracycline). The red horizontal dashed line indicates the 80% quality filtering threshold. (**B**) Chromosomal positions of the six AMR genes mapped across the 4,196,931 bp circularized genome (x-axis in Megabases, Mb). Pinhead arrows mark exact gene loci and transcription orientation. All detected genes are chromosomally encoded and non-mobilizable.

**Table 1:** Summary statistics of the *Bacillus subtilis* SNUY1 genome assembly generated using QUAST.

Value	Parameter
*Bacillus subtilis* SNUY1	Bacteria
4,196,931 bp	Total assembly length
1	Total contigs
4,196,931 bp	Largest contig
4,196,931 bp	N50
43.69%	GC content

**Table 2:** Summary of tRNA genes identified in *Bacillus subtilis* SNUY1 using tRNAscan-SE.

Result	Feature
tRNAscan-SE	Tool used
*Bacillus subtilis* SNUY1	Genome analyzed
86	Total tRNAs detected
Ala, Leu, Lys, Arg, Gly, Ser, Met, Ile, Tyr, Glu and others	Detected amino acid classes

**Table 3:** Comparative feature analysis of MAYA against existing bacterial annotation pipelines.

Feature/Capability	MAYA (This Work)	Bactopia	BAGEP	nf-Core/Bacass	BacExplorer
Workflow Engine	Snakemake	Nextflow	Bash/Python (version 3.9.25)	Nextflow	Web/Python
Per-Rule Version Locking	Yes (Conda)	Yes (Containers)	Limited	Yes (Containers)	No
Dual Input (FASTQ & FASTA)	Yes	Yes	FASTA only	FASTQ primary	FASTA primary
Integrated Secondary Metabolites (antiSMASH)	Yes	Optional/Extra	No	No	No
Plasmid Typing (MOB-suite)	Yes	External plugin	No	No	No
Multi-layered Proteome Annotation	Yes (Prokka, DIAMOND, HMMER, KofamScan)	Prokka/Bakta	Basic	Prokka/Bakta	Basic

**Table 4:** Comparison of genomic features annotated by Prokka between FASTA and FASTQ.

Feature Type	Description	FASTA Assembly Count	FASTQ Assembly Count	NCBI Assembly Count
CDS	Coding Sequences (Protein-coding genes)	4214	4313	4237
rRNA	Ribosomal RNAs (16S, 23S, 5S)	30	25	30
tRNA	Transfer RNAs	87	106	86
tmRNA	Transfer-Messenger RNA	1	1	1
Total	Annotated Genomic Features	4332	4445	4354

**Table 5:** Comparative DIAMOND functional annotation metrics.

Metric/Parameter	Assembly FASTA	Raw/Assembled Reads FASTQ
Total Sequence Hits	4135	4214
Unique Query IDs	4135	4170
Mean % Identity	99.90%	99.75%
Mean Alignment Length	295.9 aa	293.6 aa
Metric/Parameter	36.2–10,851	36.2–10,015
Median E-value	2.16 × 10^−180^	2.48 × 10^−179^

**Table 6:** Comparative AMR gene detection in FASTA and FASTQ datasets using MAYA.

Gene	FASTA Identity (Coverage)	FASTQ Identity (Coverage)
*mphK*	100.00% (100.00%)	100.00% (100.00%)
*vmlR*	100.00% (100.00%)	100.00% (100.00%)
*aadK*	100.00% (100.00%)	100.00% (100.00%)
*satA_Bsub*	100.00% (100.00%)	100.00% (100.00%)
*tet(L)*	100.00% (100.00%)	99.93% (98.47%)
*rphC*	82.42% (99.35%)	82.42% (99.35%)
*cat-TC*	—	99.42% (96.37%)

**Table 7:** Comparative MLST profile of *Bacillus subtilis* 168 from FASTA and FASTQ datasets using MAYA.

MLST Result	FASTA	FASTQ
Sequence Type	ST1	ST1
Seven-locus identity	100%	100%

**Table 8:** Biosynthetic gene cluster (BGC) predictions identified by antiSMASH in *Bacillus subtilis* 168.

Region ID	Assembly FASTA (NC_000964.3)	Read FASTQ (fastq_SRR15006059)	Biosynthetic Type/Cluster Classification
Region 1	203,941–226,894 bp	Region 5.1 (NODE_5: 27,704–50,657 bp)	sactipeptide, ranthipeptide
Region 2	356,968–422,359 bp	Region 5.2 (NODE_5: 180,730–219,129 bp) Region 6.1 (NODE_6: 134,058–159,319 bp)	NRPS
Region 3	1,149,943–1,170,746 bp	Region 1.4 (NODE_1: 497,385–519,283 bp)	terpene
Region 4	1,763,763–1,878,521 bp	Region 1.6 (NODE_1: 732,930–847,689 bp)	transAT-PKS, PKS-like, T3PKS, NRPS
Region 5	1,940,198–2,017,957 bp	Region 1.5 (NODE_1: 593,494–671,253 bp)	NRPS, betalactone
Region 6	2,092,168–2,114,066 bp	Region 1.7 (NODE_1: 1,440,708–1,461,511 bp)	terpene
Region 7	2,259,521–2,279,691 bp	Region 1.3 (NODE_1: 331,762–351,932 bp)	glycocin
Region 8	2,296,956–2,338,053 bp	Region 1.2 (NODE_1: 273,400–314,497 bp)	T3PKS
Region 9	2,515,789–2,536,679 bp	Region 1.1 (NODE_1: 74,776–95,668 bp)	terpene-precursor
Region 10	3,246,008–3,312,296 bp	Region 2.6 (NODE_2: 883,676–949,964 bp)	terpene-precursor, NRP-metallophore, NRPS
Region 11	3,593,821–3,614,567 bp	Region 2.5 (NODE_2: 574,102–594,848 bp)	CDPS
Region 12	3,826,058–3,847,669 bp	Region 2.4 (NODE_2: 340,999–362,610 bp)	sactipeptide
Region 13	3,850,668–3,892,086 bp	Region 2.3 (NODE_2: 296,835–338,253 bp)	other
Region 14	4,088,150–4,108,419 bp	Region 2.2 (NODE_2: 80,621–100,890 bp)	RRE-containing
Region 15	4,115,742–4,137,440 bp	Region 2.1 (NODE_2: 51,600–73,298 bp)	epipeptide

**Table 9:** Comparative tRNA prediction in FASTA and FASTQ datasets using tRNAscan-SE within the MAYA pipeline.

Feature	Reference Genome—FASTA	Read-Derived Assembly—FASTQ
Tool used	tRNAscan-SE	tRNAscan-SE
Dataset analyzed	*B. subtilis* 168 (NC_000964.3)	fastq_SRR15006059
Total tRNAs detected	86	114
Standard tRNAs	—	105
Pseudogenes	—	4
Undetermined tRNAs	—	5
Major amino acid classes detected	Leu, Gly, Arg, Glu, Ala, Ser, Val, Thr, Lys, Asn, and others	Arg, Leu, Gly, Ala, Val, Pro, Thr, Asn, Glu, Asp, and others

**Table 10:** In silico mobility prediction and replicon typing of plasmids/contigs using MOB-suite.

Contig ID	Size (bp)	GC (%)	Replicon Type	Conjugation Machinery (Relaxase/MPF/oriT)	Predicted Mobility	Nearest Mash Match	Closest Taxon
AC670	3404	36.28	rep_cluster_1018	Absent	Non-mobilizable	U32369	*Bacillus cereus*
novel_21d5243d86abc7218238d2dc65ced188	1666	43.64	ColRNAI_rep_cluster_1987	Absent	Non-mobilizable	CP030184	*Salmonella enterica*

**Table 11:** Summary statistics of the *Bacillus subtilis* subsp. *subtilis* genome assembly generated using QUAST.

Value	Parameter
*Bacillus subtilis* subsp. *subtilis* str. 168	Organism
4,215,606 bp	Total assembly length
1	Total contigs
4,215,606 bp	Largest contig
4,215,606 bp	N50
43.51%	GC content

**Table 12:** Comparative analysis of SNUY1 with a published genome.

Published Genome Paper	MAYA Pipeline	Feature
4,196,931	4,196,931	Genome size (bp)
43.7	43.69	G + C content (%)
1	1	Number of contigs
4,196,931	4,196,931	N50 (bp)
4272	4196	CDS
86	86	tRNA
30	30	rRNA
5 ncRNAs including tmRNA	1 tmRNA	tmRNA/ncRNAs
100	100	BUSCO completeness (%)

## Data Availability

The sequencing data used in this study are publicly available under accession CP199918 in the National Center for Biotechnology Information (NCBI; https://www.ncbi.nlm.nih.gov). The MAYA Snakemake pipeline, including all rule definitions and per-rule Conda environment specification files, is hosted on GitHub at https://github.com/Bharat-Genome-Database-BGDB/MAYA-Pipeline (accessed on 9 September 2026) under the MIT License. The repository is currently private and will be made publicly available prior to publication; access for verification by reviewers or editors can be provided upon request in the meantime.

## Supplementary Materials

The following supporting information can be downloaded at: https://www.mdpi.com/article/10.3390/ijms27209188/s1.
